# Ultrasound-Guided Popliteal Nerve Block in a Patient with Malignant Degeneration of Neurofibromatosis 1

**DOI:** 10.1155/2012/753769

**Published:** 2012-05-09

**Authors:** Arjun Desai, Brendan Carvalho, Jenna Hansen, Jonay Hill

**Affiliations:** Department of Anesthesia, H3580, Stanford University School of Medicine, Stanford, CA 94305, USA

## Abstract

A 41-year-old female patient with neurofibromatosis 1 presented with new neurologic deficits secondary to malignant degeneration of a tibial lesion. Ultrasound mapping of the popliteal nerve revealed changes consistent with an intraneural neurofibroma. Successful popliteal nerve blockade was achieved under ultrasound guidance.

## 1. Introduction

Neurofibromatosis 1 (NF) or von Recklinghausen disease is a systemic genetic disorder that commonly presents with cutaneous neurofibromas, café-au-lait spots, and hallmark plexiform neurofibromas [1]. NF presents several difficulties for anesthesiologists including potential airway difficulty, abnormal spinal anatomy, and peripheral neurofibromas [2].

The role of CT and MRI are well established for mapping anatomical variations in airway and spinal regions in patients with neural sheath tumors. Recently, ultrasound (US) has become a useful adjunct diagnostic modality in occult NF [3]. With widespread use of US in regional anesthesia, sonographic characteristics of neurofibromas are increasingly described in the literature [4]. However, there is a lack of information regarding the efficacy and safety of providing regional anesthesia in the setting of peripheral nerve sheath tumors. We present a case of successful popliteal nerve blockade under ultrasound guidance in a patient with NF by administering local anesthetic proximal to a lesion consistent with intraneural neurofibromatosis.

## 2. Case Report

A 41-year-old Caucasian female patient with a past medical history significant for NF presented for open biopsy of her left tibia. The patient detailed a one and a half year progressive weakness and pain in her left ankle. Initial plain film radiographic examination in August 2010 showed a 3 × 1.5 × 2 cm destructive lesion of the cortical bone in the distal tibia. MRI analysis of the lesion in September 2010 showed a larger enhancing 5.7 × 4.6 cm lesion. At that time the patient underwent Tru-Cut soft tissue biopsy of the mass with pathology confirming a nerve sheath tumor. However, there was insufficient tissue to determine malignancy and further tests were required. After a brief loss to follow up for insurance and financial difficulties, another MRI examination in January 2011 revealed an even larger 6.5 × 5.1 cm T2 hyperintense lesion eroding through the tibia with a 2.4 cm proximal periosteal extension. The patient's condition had also deteriorated, and she now required crutch support for ambulation. She reported decreased sensation to cold, light touch, and pinprick over the left first and second toes. Concern for malignant degeneration of her NF disease in the left ankle warranted a more extensive open biopsy for definitive diagnoses.

Preoperative anesthetic evaluation revealed no other significant medical problems, no known drug allergies, no prior general anesthetics, and a family history of pseudo-cholinesterase deficiency. She had several previous cutaneous nodule excisions for cosmesis completed under a combination of local anesthesia and sedation at outside institutions. The patient was prescribed temazepam (Restoril, Mallinckrodt Pharmaceuticals Group, MO) orally for insomnia as necessary, as well as hydrocodone-acetaminophen (Vicodin, Abbott Laboratories, IL) and ibuprofen (Motrin, McNeil Consumer Healthcare Division, PA) orally as required for ankle related pain. On general examination, she had multiple cutaneous neurofibromas diffusely distributed throughout her body. Her BMI was 29 and her airway examination was unremarkable. 

### 2.1. Popliteal Nerve Regional Anesthetic Blockade

Prior to the nerve blockade, standard monitors (noninvasive blood pressure cuff, pulse oximeter, and 3-lead ECG) were applied. Supplemental oxygen (3 L/minute) was administered via nasal cannula. Adequate sedation was achieved with intravenous midazolam 2 mg (Versed, Roche, NY), and fentanyl 150 mcg after timeout was completed. The patient was placed in supine position with left foot elevation and left knee flexion, and the skin sterilized with ChloraPrep (Cardinal Health, OH).

**Figure 1:**
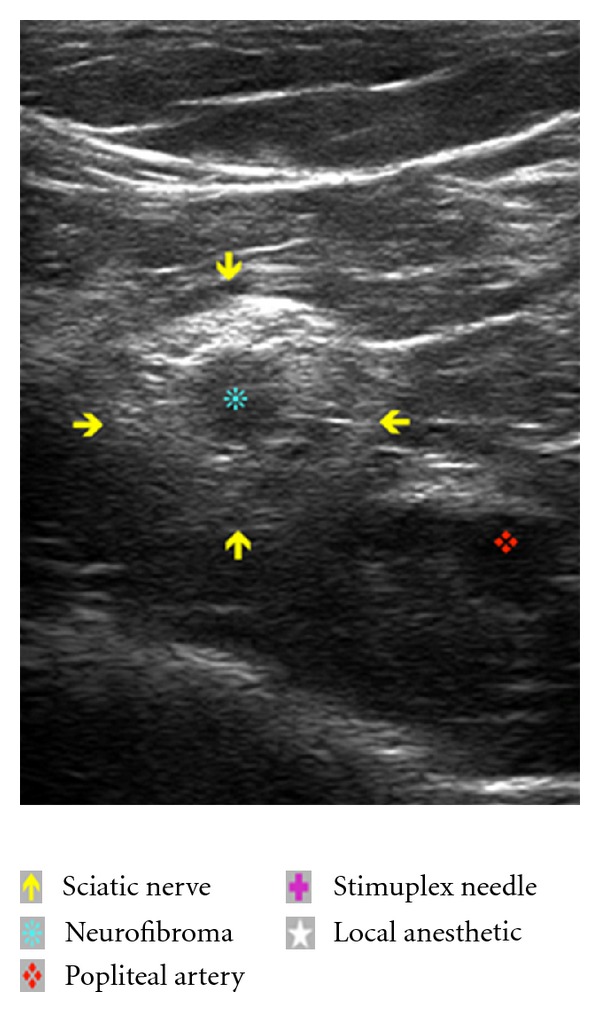
Ultrasound image of popliteal fossa including the sciatic nerve with intraneural neurofibroma and popliteal artery.

**Figure 2:**
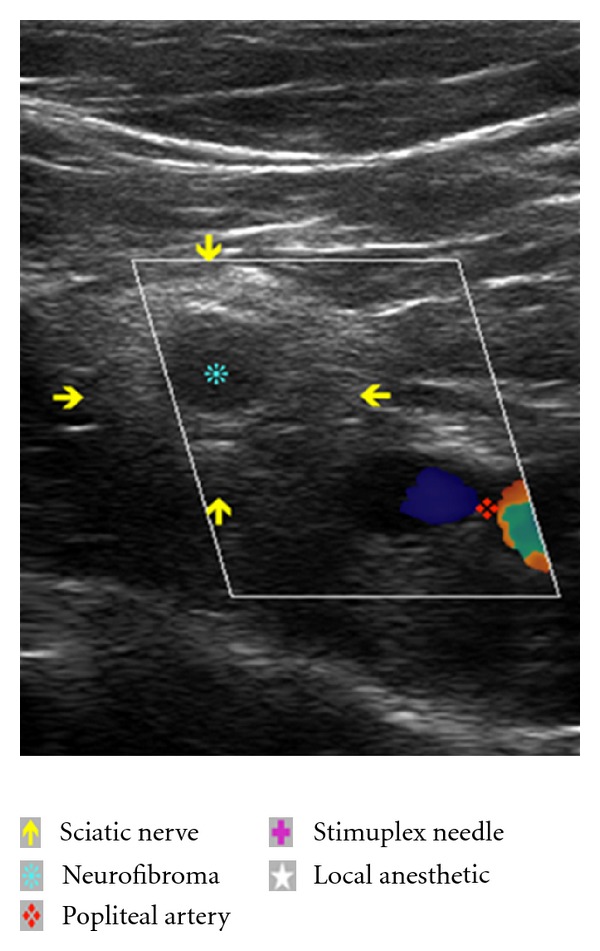
Ultrasound image with doppler flow analysis of popliteal fossa including the sciatic nerve with intraneural neurofibroma and popliteal artery. Doppler analysis correctly identifies blood flow in the popliteal artery and lack of flow in the intraneural inclusion of the sciatic nerve.

**Figure 3:**
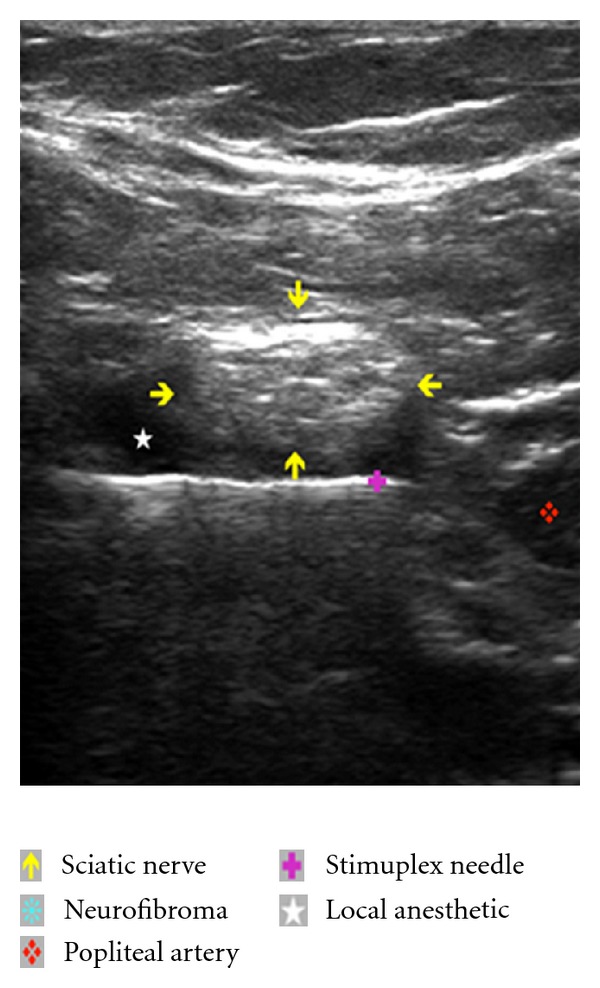
Ultrasound image of the popliteal fossa including the sciatic nerve with no intraneural inclusion, perineural infiltration of local anesthetic, Stimuplex needle, and popliteal artery.

 The US transducer (linear, 12-3 MHz, L12-3 Phillips) was placed transversely at the inferior aspect of the popliteal fossa. Cephalad and caudad orientation of the probe demonstrated relevant anatomy including the popliteal artery, vein, sciatic nerve, and its bifurcation into the common peroneal and tibial nerves. Of specific interest, sonographic “target signs” [5] consistent with previous descriptions of intraneural fibromas [6] were demonstrated in the sciatic, common peroneal, and tibial nerves [7]. The lesions appeared solitary, ovoid and contiguous with the nerve, had a hypoechoic echotexture with well-defined margins, and demonstrated subtle distal acoustic enhancement (Figure 1). With popliteal artery in view, we were able to demonstrate true and faux (neurofibroma) arterial structures using doppler analysis (Figure 2). Tracing the path of the sciatic nerve proximally, we identified a portion of the nerve with no neurofibromatous changes on ultrasound. We also carefully selected an area of the inferior thigh void of cutaneous neurofibromas for needle insertion. The skin was infiltrated with 2 mL 2% lidocaine, and a 10 cm 22-gauge Stimuplex needle (B. Braun, PA) was utilized. A real-time view of the Stimuplex needle tip, popliteal artery, and sciatic nerve was maintained at all times during the block. A total of 30 mL 0.5% ropivacaine (APP Pharmaceuticals, IL) with 1 : 400.000 epinephrine was injected with intermittent negative aspiration for blood. The local anesthetic spread circumferentially around the sciatic nerve as noted on ultrasound examination (Figure 3). The patient reported no paresthesias or discomfort during the procedure.

Within ten minutes, the patient reported subjective numbness, warmth, and heaviness of her left calf and foot. On examination she had complete sensation loss to sharp touch and cold in the sciatic distribution of her left lower extremity. Intraoperatively, sedation was provided with midazolam 2 mg IV followed by a propofol infusion (25–50 mcg/kg/min). She maintained spontaneous ventilation via laryngeal mask airway. The patient did not respond to the incision and remained hemodynamically stable for the duration of surgery. She received ondansetron 4 mg (Zofran, GlaxoSmithKline, NC) intravenously twenty minutes prior to the completion of surgery for nausea prophylaxis.

The patient was alert, awake, and oriented in the postanesthesia care unit. She complained of no pain (verbal pain score 0/10) and had residual sensory loss and motor weakness of 1/5 in the left lower extremity. The patient denied symptoms of nausea and was discharged home later that afternoon. On post-operative day one, the patient was contacted at home via telephone for followup. The patient stated baseline motor and sensory functions returned approximately eight hours after the operation. Her pain was adequately addressed at home with hydrocodone-acetaminophen (Vicodin, Abbot Laboratories, IL) orally every six hours as needed. Her pain at rest was 1-2/10, and with activity increased to 3-4/10. She had no hematoma or tenderness at the site of the regional blockade. The patient reported an overall favorable anesthetic and postoperative pain experience.

## 3. Discussion

NF poses many potential challenges to the performance of general, neuraxial, and regional anesthesia [8]. Anesthesiologists must tailor their anesthetic technique on a perpatient basis, balancing the risks and benefits of specific procedures in NF patients.

Neuraxial (epidural and spinal) anesthesia may be relatively contraindicated in many patients with NF. Clinically silent neurofibromas may involve spinal cord and nerve roots in up to 40% of patients [9]. Risks associated with neuraxial anesthesia range from hematoma, paralysis, or even death with acute spinal decompression [10]. Despite these dangers, the literature shows that preintervention anatomic mapping via CT and MRI can lead to safe and effective clinical interpretations and outcomes [7, 11].

Although neuraxial anesthesia has been studied extensively in NF patients, we found limited published reports of outcomes for regional anesthesia procedures [4]. There are however increasing sonographic descriptions of neurofibromas in the regional anesthesia literature. In addition, real-time US imagery allows accurate lesion localization and may possibly reduce the risks. In this paper we were able to demonstrate previously described ultrasonographic characteristics of neurofibromas. In particular, we demonstrate the “target sign” [5, 12] and replicate images consistent with published descriptions of solitary, ovoid, and hypoechoic intraneural lesions with well-defined margins and subtle distal acoustic enhancements [5–7]. The hypoechoic foci are believed to be caused by collagen deposits [13]. Beggs et al. also details subtle differences between neurilemomas (where the nerve travels peripherally to the lesion) and neurofibromas (where the nerve runs into the middle of the lesion). Building on these concepts, the use of US as both a diagnostic tool for NF and a therapeutic guide for interventional procedures is becoming increasingly evident.

Establishing the sonographic characteristics of neural sheath tumors enabled us to diagnose the NF lesions and differentiate them from other soft tissue structures such as lymph nodes [14] (Figure 2). US also allowed us to insert the needle in an area void of neural tumors. The doppler function highlighted vasculature structures, differentiating vascular from relevant nerve anatomy or tumor structures (Figure 1).

Despite well-described sonographic details of peripheral nerve sheath tumors, the efficacy and outcomes of regional blockade in patients with NF1 is limited [4]. Manickam et al. [14] describe the utility of a thorough sonographic survey. After defining an intraneural lesion with US in their patient, the team confirmed multiple similar lesions along the proximal course of the nerve. Their patient however chose to avoid regional anesthesia and had an uncomplicated general anesthetic. Rocco and Rosenblatt [4] reported performing successful regional blockade in a patient with an intraneural lesion in the popliteal fossa. The authors performed this block after a detailed sonographic survey to avoid direct contact with the tumor.

In our case, we used US to identify lesions and provide sonopathology of the NF tumors. We then performed a detailed sonographic survey to define lesion-free nerve that would minimize injury to the neurofibroma and reduce any associated risks. The block efficacy appeared to be unaffected by the NF tumors. The onset, duration, and resolution of regional anesthesia in our NF patient were as expected in a healthy patient.

In conclusion, we report successful popliteal nerve block using US guidance in a patient with NF. US guidance allows a unique opportunity to visualize and therefore avoid puncture of lesions in patients with NF and other types of neuralsheath tumors. Although this paper demonstrates that regional anesthesia can be offered to and successfully performed in patients with NF, more research is necessary to show efficacy and safety of peripheral nerve blockade in these patients. Potential issues that may be unique to NF patients include alterations in nerve conduction, spread of local anesthetic, and onset and duration of blockade. We recommend that any decision to use a regional anesthetic technique should be individualized. Further studies of the safety and efficacy of regional anesthesia in this patient population are necessary to establish consensus for routine practice of a regional anesthetic technique in this setting.
